# Reviewed article: Correia JVP, Silva-Oliveira F, Ferreira RC, Zarzar PMPA. Characterization, frequencies and comparison of child abuse reporting rates: a comparative study, Brazil, 2011-2021. Epidemiol Serv Saude. 2025:34;e20240545

**DOI:** 10.1590/S2237-96222025v34e20240545.b

**Published:** 2025-10-10

**Authors:** Laura Inês Gomes Maricato

**Affiliations:** 1 Instituto Federal de Alagoas, Macéio, AL, Brazil Instituto Federal de Alagoas Macéio AL Brazil

## Peer review B

## Questionnaire

Does the manuscript contain new and significant information to justify publication? Yes

Does the Abstract (Summary) clearly and accurately describe the content of the article? Yes

Is the problem significant and concisely stated? No

Are the methods described comprehensively? No

Are the interpretations and conclusions justified by the results? Yes

Is adequate reference made to other work in the field? Yes

## Manuscript Structure

Length of article is: Adequate

Number of tables is: Too many

Number of figures is: Too few

Please state any conflict(s) of interest that you have in relation to the review of this paper. None

Interest: Good

Quality: Below Average

Originality: Good

Overall: Good

Recommendation: Major Revision

## Comments

Caro autor,

O artigo traz a importância para criação de novas Leis e enfatiza a capacitação dos profissionais da saúde para casos de violência infantil no Brasil. No entanto, gostaria de sugerir algumas melhorias para fortalecer ainda mais o estudo. Os métodos utilizados precisam ser mais detalhados para garantir replicabilidade, e algumas das referências podem ser atualizadas para refletir estudos mais recentes (se aplicável). Na discussão dos resultados poderia se beneficiar de uma análise mais aprofundada das implicações práticas para essas crianças. Espero que essas recomendações possam enriquecer ainda mais o seu trabalho.

Considere antes da metodologia descrever seus objetivos gerais e específicos (se aplicável) e se achar necessário considerar adaptar elementos do PICO para ajudar a estruturar a pergunta). Não encontrei informações sobre o objetivo do estudo além do resumo.

Desfechos, quais são? Existem? Deixá-los de forma mais clara.

Revise os métodos, descreva com mais detalhes incluindo o processo de coleta e análise de dados para que outros pesquisadores possam replicar o estudo. Explique como os vieses foram minimizados e como a validade interna foi garantida. Processo foi realizado em duplicata?

As tabelas precisam ser revisadas para garantir clareza, precisão e evitar redundância com o texto. Não encontrei a chamada para as tabelas 3 e 4 e nem para o gráfico 1.

Sugiro trazer para a discussão a validade externa do estudo.

Inclua um cálculo de poder para demonstrar que a amostra é suficiente para detectar um efeito clinicamente relevante. Para que a amostra seja representativa e o tamanho suficiente para gerar resultados estatisticamente robustos.

O texto precisa ser revisado para garantir clareza, precisão e objetividade na linguagem. As ideias devem ser apresentadas de forma organizada e lógica. Verifique erros gramaticais, de formatação e de estilo, incluindo o uso de letras maiúsculas. Não há espaçamento entre os parênteses e as palavras, por exemplo: “família(10-12)”.

Verifique se as referências citadas são relevantes, atuais e apoiam as afirmações feitas no texto. Inclua referências importantes que podem ter sido omitidas. Encontrei alguns erros na formatação das referências que não seguem as normas, por exemplo: “1993;17(2):198-209” (deve ser junto e sem espaços) foi encontrado algumas referências com espaçamento. Algumas referências estão faltando link ou DOI.

Página 1:

O primeiro parágrafo na introdução precisa fazer conexão com o segundo parágrafo. Ficou disperso o assunto do segundo parágrafo da introdução. Considere adaptar o segundo parágrafo da introdução de forma que fique mais claro e fluído para leitura. Como sugestão, talvez, remover o “estudo”...

Na seguinte frase “Portanto, o setor da saúde “constitui uma encruzilhada para onde se convergem os desfechos dos maus-tratos infantis, pela pressão que as vítimas exercem sobre os serviços” - Sugestão, nessa frase explique ou pontue o que é/são a(s) “pressão(ões)” que as vítimas exercem sobre os serviços e quais serviços são esses ou podem ser?

No terceiro parágrafo, “Estudos indicam que o domicílio é o local mais comum de ocorrência, tendo como agressores pessoas próximas à criança, geralmente da própria família(10-12).” Sugestão melhorar para melhor fluidez.

Quinto parágrafo da introdução: “...Os dados notificados pelo SINAN contribuem para compreender a magnitude e perfil dos casos...” Sugestão, substituir por “...Os dados notificados pelo SINAN contribuem para compreender a magnitude e perfil da violência infantil no país...” Dessa forma evita-se a repetição de palavras na mesma frase.

Ainda no quinto parágrafo da introdução: “Análises dos dados do SINAN podem também demonstrar o perfil dos atendimentos relacionados a esses agravos no sistema de saúde nacional, além de evidenciar ainda a necessidade de abordar lacunas na notificação nesse sistema de informação(18).” Essa frase está em sobreposição com a anterior, rever.

Sexto parágrafo da introdução: “Alguns estudos analisam dados de violência infantil notificados ao SINAN, mas, principalmente, com dados estaduais ou municipais(19,20)”... Sugestão reescrever para melhor fluidez.

Recomendo, trazer na introdução o que é o SINAN ao invés de explicá-lo na metodologia.

No tópico “Em participantes” não caberia explicar os critérios de exclusão? E na frase “A população do estudo compreendeu” sugiro melhorar talvez para “a população elegível para o estudo foram os casos”...

Na frase “A ficha de notificação é preenchida pelos profissionais de saúde nas unidades para cada indivíduo quando acontece a suspeita da ocorrência de problema de saúde de notificação compulsória. O instrumento deve ser encaminhado pelos serviços públicos e privados aos serviços responsáveis pela informação ou vigilância epidemiológica das Secretarias Municipais, que devem repassar semanalmente os arquivos para as Secretarias Estaduais de Saúde.” Verifique melhorar estrutura desse parágrafo para melhor fluidez, clareza e objetividade.

Em resultados: “Para crianças de 5-9 anos, observou-se também maiores taxas entre meninas para os casos notificados de violência sexual e psicológica em todos os anos.” Mesma coisa sendo dita mais de uma vez, no mesmo parágrafo e encontrei na discussão ... rever o texto e apenas manter uma vez escrita.

Em discussão: “A análise da proporção de notificações por raça/cor da pele das crianças, demonstra maiores valores para crianças pardas. Considerando as crianças negras (pardas e pretas), essa proporção chega a 52,9%. Estudos nacionais(19,20) e internacionais(22,23). No entanto, alguns estudos brasileiros apresentam uma situação diferente, apontando as crianças brancas como proporcionalmente mais afetadas(10,24). Considerando que as crianças negras representam aproximadamente 56,7% da população infantil brasileira, conforme dados do Instituto Brasileiro de Geografia e Estatística, essa discrepância levanta a possibilidade de sub-identificação e/ou subnotificação de violências entre essas crianças ou então elas podem ter menos acesso aos serviços de saúde, o que poderia impactar na notificação. Também podemos nos questionar se os profissionais, devido ao racismo estrutural, não estejam invisibilizando alguns casos de violência contra crianças negras. Portanto, medidas eficazes de prevenção e controle da violência infantil devem ser complementadas por políticas amplas de combate ao racismo estrutural.” > revisar o parágrafo, escrever de forma mais concisa. Desse modo fica confuso para leitura.

“Outro ponto relevante em relação à negligência que pode influenciar na decisão de notificar é a falsa percepção, entre profissionais de saúde, de que a negligência poderia ser uma forma de abuso com menor potencial para causar danos” > nessa frase, sugiro melhorar a escrita para melhor fluidez.

Em discussão:

Como sugestão acredito ser interessante trazer informações de outros artigos, o que trazem como solução? Para evitar ou impedir casos de violência infantil. Como estão gerenciando esses ocorridos? O que os outros países têm se adiantado nesse assunto? Existem recursos? Quais são as medidas?

